# Abnormal Situation of the Anus with Intestinal Calculi

**Published:** 1870-04

**Authors:** B. C. Brett

**Affiliations:** Broadhead, Wisconsin


ARTICLE V.—*Abnormal situation of the Anus with Intestinal Calculi.* By B. C. BRETT, M.D., Broadhead, Wisconsin.

HEREWITH I transmit the report of a case which may be interesting to the medical gentlemen who read your journal, not because there is anything striking in an operation which the good sense of any physician would have suggested, but because of the rarity of circumstances which would call for such an operation.

I was called, December 10, 1869, to see B. G., a little girl, ten years old. On arriving, I learned from her mother the following history:

She was congenitally deformed ; the anus being too small and situated too far forward. Her health was always poor, owing, as she thought, to the heavy doses of cathartics which she was obliged to take. When she was two years old she discovered some plum seeds in the rectum, and, with a bent wire, succeeded in bringing out some of them ; but others remaining, had seemed to increase in size until their presence became a serious hindrance to defecation. The dejections were never larger than a clay pipe-stem.

On examination, I found an anal aperture a half-inch in diameter, when fully distended, communicating with the rectum by a canal three-fourths of an inch long, and situated close to the posterior commissure of the labia majora. In fact, the perineum consisted of simply a continuation of the recto-vaginal septum of a uniform thickness from above downwards. The normal point for the anus was marked by a faint red line and slight depression about an inch in rear of this opening. Having passed a sound into the rectum, I found it unusually large, it having been subjected to almost constant distention for several years. At about four inches from the orifice I detected a concretion, which I brought down by using the sound, bent in the shape of a hook. Having grasped the stone with a pair of slender polypus forceps, I found it too large to remove except by crushing, or by enlargement of the aperture, which latter operation, in consideration of the abnormal size and situation of the anus, seemed advisable. Accordingly, having put the patient under chloroform, the posterior wall of the canal was incised to the depth of over a half inch, when the stone was removed without difficulty. I also detected and removed three others. To prevent union of the cut surfaces, and thus insure permanent enlargement of the anus, the finger has been used as a bougie once a day. The patient is now walking about, retains her fæces well, has an easy and free evacuation each day, and enjoys better health than ever before.

The largest of the stones removed measured three and a half inches in circumference. They were all ovoid in form, and nearly equal in size. Having divided one of them, I find it composed of concentric layers of what appears to be carbonate and phosphate of lime, having a plum seed for a central nucleus. The probability is that these seeds which, in a well formed child, would have passed readily out, in this case had remained in the rectum, gradually increasing in size, for these eight years.

To what extent this patient will experience the need of a better perineum if she lives to become a mother, is an interesting query.

